# Supplementary material for: A fault-intrusion-tolerant system and deadline-aware algorithm for scheduling scientific workflow in the cloud
Source: PeerJ Comput Sci. 2021 Nov 2;7:e747. doi: 10.7717/peerj-cs.747 (PMC8576554; doi:10.7717/peerj-cs.747)
Supplement: Supplemental Information 6 [file peerj-cs-07-747-s006.zip › Myproject1.1/docs/org/cloudbus/cloudsim/examples/power/package-summary.html]

org.cloudbus.cloudsim.examples.power


JavaScript is disabled on your browser.


- Overview
- Package
- Class
- Use
- Tree
- Deprecated
- Index
- Help

- Prev Package
- Next Package

- Frames
- No Frames

- All Classes

# Package org.cloudbus.cloudsim.examples.power

- Class Summary

  | Class | Description |
  | --- | --- |
  |  |  |
  | --- | --- |
  | Constants | If you are using any algorithms, policies or workload included in the power package, please cite the following paper: Anton Beloglazov, and Rajkumar Buyya, "Optimal Online Deterministic Algorithms and Adaptive Heuristics for Energy and Performance Efficient Dynamic Consolidation of Virtual Machines in Cloud Data Centers", Concurrency and Computation: Practice and Experience (CCPE), Volume 24, Issue 13, Pages: 1397-1420, John Wiley & Sons, Ltd, New York, USA, 2012 |
  | Helper | The Class Helper. |
  | RunnerAbstract | The Class RunnerAbstract. |

- Overview
- Package
- Class
- Use
- Tree
- Deprecated
- Index
- Help

- Prev Package
- Next Package

- Frames
- No Frames

- All Classes
